# Understanding the functional difference between growth arrest-specific protein 6 and protein S: an evolutionary approach

**DOI:** 10.1098/rsob.140121

**Published:** 2014-10-22

**Authors:** Romain A. Studer, Fred R. Opperdoes, Gerry A. F. Nicolaes, André B. Mulder, René Mulder

**Affiliations:** 1 European Molecular Biology Laboratory-European Bioinformatics Institute (EMBL-EBI), Wellcome Trust Genome Campus, Hinxton, Cambridge CB10 1SD, UK; 2 Laboratory of Biochemistry, de Duve Institute and Université catholique de Louvain, Brussels 1200, Belgium; 3 Department of Biochemistry, Cardiovascular Research Institute Maastricht, Maastricht University, Maastricht, The Netherlands; 4 Department of Laboratory Medicine, University Medical Centre Groningen, Groningen, The Netherlands

**Keywords:** protein S, growth arrest-specific protein 6, evolution

## Abstract

Although protein S (PROS1) and growth arrest-specific protein 6 (GAS6) proteins are homologous with a high degree of structural similarity, they are functionally different. The objectives of this study were to identify the evolutionary origins from which these functional differences arose. Bioinformatics methods were used to estimate the evolutionary divergence time and to detect the amino acid residues under functional divergence between *GAS6* and *PROS1*. The properties of these residues were analysed in the light of their three-dimensional structures, such as their stability effects, the identification of electrostatic patches and the identification potential protein–protein interaction. The divergence between *GAS6* and *PROS1* probably occurred during the whole-genome duplications in vertebrates. A total of 78 amino acid sites were identified to be under functional divergence. One of these sites, Asn463, is involved in *N*-glycosylation in GAS6, but is mutated in PROS1, preventing this post-translational modification. Sites experiencing functional divergence tend to express a greater diversity of stabilizing/destabilizing effects than sites that do not experience such functional divergence. Three electrostatic patches in the LG1/LG2 domains were found to differ between GAS6 and PROS1. Finally, a surface responsible for protein–protein interactions was identified. These results may help researchers to analyse disease-causing mutations in the light of evolutionary and structural constraints, and link genetic pathology to clinical phenotypes.

## 2. Introduction

Growth arrest-specific protein 6 (GAS6, MIM# 600441) and protein S (PROS1, MIM# 176880) are homologous vitamin K-dependent proteins [1]. Whereas GAS6 is the main ligand for receptor tyrosine kinase Tyro3, Axl and Mer (TAM), several lines of evidence have shown that PROS1 also interacts with Tyro3 and Mer, but with a high degree of species specificity [2]. No interactions between PROS1 and Axl have been reported. PROS1 functions as a cofactor for activated protein C (APC) in the proteolytic degradation of activated coagulation factors Va (FVa) and VIIIa (FVIIIa) [3,4]. Recently, PROS1 has also been identified to function as a cofactor for tissue factor pathway inhibitor (TFPI), accelerating the inhibition of activated factor Xa (FXa) [5]. GAS6 and PROS1 have been associated with a wide variety of conditions and disorders, including thrombosis [6,7], systemic lupus erythematus [8,9], kidney disorders [10,11], sepsis [12,13], cancer [14,15], pregnancy [16], infections such as human immunodeficiency virus [17] and during the use of oral contraceptives [18]. Interestingly, both proteins exhibit different expression profiles. Contrary to PROS1, GAS6 is not expressed in the liver, and its concentration in human plasma is almost 1500-fold less than that of PROS1 (0.22 versus 346 nmol l^−1^) [19,20].

GAS6 and PROS1 show a high degree of similarity, both in module organization and at the amino acid level. GAS6 is 721 amino acids long (the isoform 2 has a length of 678 amino acids) and PROS1 is 676 amino acids long. Both are multimodular proteins with an N-terminal region containing the γ-carboxyglutamic acid (GLA) domain, which is formed after the post-translational modification of glutamic acid [21]. The GLA domain is followed by a thumb loop, four sequentially arranged epidermal growth factor-like (EGF) domains and two laminin G (LG) domains that make up the sex hormone-binding globulin (SHBG). The SHBG-domain of GAS6 is required for its interaction with the Axl receptor [22]. The binding site for complement component C4-binding protein (C4BP) is contained in both LG domains within the SHBG-domain of PROS1 [23–32], whereas the LG domains of PROS1, and in particular LG2, were shown to be indispensable for expression of the anticoagulant activities in the APC-catalysed inactivation of FVa and FVIIIa [33,34]. The LG2 domain of PROS1 also seems to contain a binding site for FVa [35]. Recently, the LG1 domain of PROS1 was shown to be essential for binding and enhancement of TFPI [36].

In plasma, approximately 60% of the total amount of PROS1 is bound to C4BP, while the remaining 40% circulates free and functions as a cofactor for APC. It has recently been suggested that residues within the GLA and EGF1 domains of PROS1 act cooperatively for its APC cofactor function [37]. The PROS1-binding site on C4BP is contained within the first short consensus repeat (SCR) of its beta-chain [38–41]. SCR2 contributes to the interaction of SCR1 with PROS1 [42–44].

As GAS6 and PROS1 homologues share a common ancestor and have retained overall structural similarities, why are they functionally different? For example, both GAS6 and PROS1 are post-translationally modified through *N*-linked glycosylation (addition of a *N*-acetyl-d-glucosamine to an asparagine), but at different positions, suggesting a potential shift in function. The availability of whole-genome data has enabled scientists to address such questions through bioinformatic approaches. GAS6 and PROS1 are paralogous genes that were separated during a duplication event, probably during the two rounds of whole-genome duplication at the beginning of vertebrate evolution. Gene duplication followed by speciation provides opportunities for the creation of novel genetic content [45–47]. The replacement (or substitution) rate of amino acids in proteins can be accelerated or decelerated, depending on the functional constraints and the selective advantage of these new mutations [48]. Advantageous mutations become ultimately fixed in the population. Such functional divergence at the level of amino acids between homologous genes can be classified into two types (Type I or Type II) of functional divergence [49,50]. Type I is characterized by amino acid patterns that are highly conserved in one group of sequences (clade) but highly variable in the other. On the other hand, Type II represents amino acid patterns that are highly conserved in one group of sequences (clade) and also conserved in the other group of sequences, but for a different amino acid. Sites detected under either Type I or Type II of functional divergence could explain the functional differences between groups of sequences (orthologues or paralogues) [51].

In this context, we used an evolutionary approach to (i) identify the gene duplication and the subsequent evolution that lead to the formation of GAS6 and PROS1, (ii) identify amino acid regions that are responsible for functional divergence between GAS6 and PROS1, and (iii) elucidate the structural impact of these regions on the GAS6 and PROS1 protein structures.

## 3. Material and methods

### 3.1. Data collection

Homologous protein sequences were collected by running BlastP searches of the human PROS1 sequence (Uniprot ID: P07225) against the UniProtKB/Swiss-Prot database (www.uniprot.org). These retrieved sequences were aligned using the L-INS-i algorithm from MAFFT (v. 7.113b), a multiple sequence alignment (MSA) program [52]. The dataset is composed of 32 sequences with 314 sites. The graphical rendering of the alignment using Jalview 2.8 [53] is provided as electronic supplementary material, figure S1. Pairwise percentage identities were calculated using ClustalX.

### 3.2. Phylogenetic analyses

**Figure 1.**
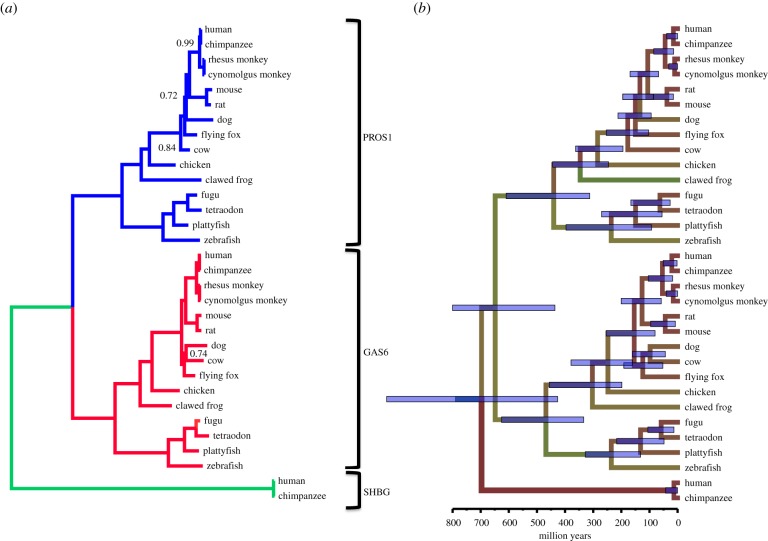
Phylogenetic consensus tree with and without molecular clock of GAS6, PROS1 and SHBG sequences. (*a*) Tree without a molecular clock model. The GAS6 clade is coloured in red, the PROS1 clade is in blue and the SHBG clade is in green. Values at the nodes indicate posterior probabilities. Only values different from 1.00 are indicated. The lengths of the axes are proportional to the estimated number of mutations per site. (*b*) Phylogenetic tree under a relaxed clock model. The tree topology is the same as that of the tree in panel (*a*). The estimated times of divergence of the more important nodes are indicated in electronic supplementary material, table S1. The blue error bars at the nodes represent the 95% confidence limits.

Phylogenetic analyses producing trees reflecting the evolutionary history of this family were carried out using three different methods: (i) a neighbour-joining (NJ) distance matrix tree with exclusion of regions containing insertions and deletions, and correction for multiple substitutions with 1000 bootstrap samplings, created using the Tree option of ClustalX; (ii) a maximum-likelihood (ML) analysis with 100 bootstrap samplings using the JTT evolutionary substitution model with gamma rate distribution carried using with the program PhyML [54]; and finally, (iii) a phylogenetic tree inferred by Bayesian analysis using the program MrBayes v. 3.2 [55]. The model using the JTT substitution matrix and a gamma rate distribution with four substitution rate categories was the best-fitting model to our data. To estimate Bayesian posterior probabilities, Markov chain Monte Carlo (MCMC) chains were run for 100 000 generations and sampled every 100 generations (burn-in: 25%). The resulting tree was rooted using mid-point rooting (figure 1; electronic supplementary material, table S1). Strict and relaxed molecular clock models were applied to the same dataset running, respectively, 100 000 and 400 000 generations (MrBayes). The molecular clock was time calibrated as follows: from the divergence times of various pairs of taxa obtained from the TimeTree web resource (http://www.timetree.org/) [56] the clock rates, in substitutions per site per Myr, were estimated and an average clock rate was calculated. Best results were obtained with the relaxed clock model.

### 3.3. Identification of amino acids under functional divergence

For the identification of functional divergence, the original dataset was limited to only GAS6 and PROS1 sequences. This resulted in a total of 30 sequences, from a GAS6 and a PROS1 clade with 15 sequences each. Amino acid sites under potential functional divergence have been identified by using three methods from two different packages: BADASP [57] and FunDi [58].

BADASP is a package to detect both Type I and Type II of functional divergence [57]. A score is given to each position on the multiple alignment on the probability to be associated with Type II and/or Type I, according to a threshold. A previous simulations study estimated this threshold to be 3.5 [59]. In this previous study, we generated alignments composed of random sites, under a nearly neutral process. We then computed the BADASP score for each site and defined the 99th percentile based on the distribution of these scores. This percentile corresponds to a score of 3.5, which we used as our threshold. It means that we tolerate 1% of false positive [59]. Type I sites are further divided into Type Ia (residues conserved in PROS1 and divergent in GAS6) and Type Ib (residues divergent in PROS1 and conserved in GAS6; table 1).

**Table 1.** Sites identified to be under functional divergence between GAS6 and PROS1. Functional divergence analysis was performed using three different methods: FunDi (FD) [59], BADASP (B) [57] and Selectome (PS) [62,63]. BADASP is a package to detect both Type I and Type II of functional divergence. Type I sites are divided into Ia (amino acid conserved in PROS1 and divergent in GAS6) or Ib (amino acid conserved in GAS6 and divergent in PROS1). FunDi aims to detect sites under functional divergence, independent of whether they belong to Type I or Type II of functional divergence. For GAS6, numbering is based on isoform 1 or 2 (between brackets). Overall, the methionine encoded by the translation initiation site is numbered as residue 1.

GAS6	AA	PROS1	AA	methods	GAS6	AA	PROS1	AA	methods
37 (37)	E	31	Q	B_Ib	384 (341)	Q	345	D	B_Ia
51 (51)	Q	44	S	PS	405 (362)	N	366	E	B_Ia, B_Ib
60 (60)	H	53	N	B_II	415 (372)	P	376	D	B_II, PS
97 (97)	N	90	R	B_Ia, B_Ib	423 (380)	Q	384	N	B_Ib
98 (98)	K	91	S	B_Ia, B_II	432 (389)	R	393	H	FD, B_Ib
100 (100)	G	93	Q	B_II	435 (392)	V	396	S	FD, B_II, PS
102 (102)	P	103	S	B_Ia	445 (402)	K	406	D	B_Ib
105 (105)	K	106	A	B_Ib, B_II	448 (405)	V	409	K	FD, B_Ib
106 (106)	N	107	Y	B_Ib	455 (412)	P	416	P	B_Ia
110 (110)	A	111	R	FD, B_Ia, B_II	456 (413)	E	417	E	B_Ib
114 (114)	Q	115	N	B_Ia	457 (414)	R	418	N	B_Ia
123 (123)	N	124	L	B_Ia, B_Ib	463 (420)	N	424	K	B_II
134 (134)	Q	135	K	B_Ib	465 (422)	T	426	Y	FD
136 (136)	L	137	G	FD	471 (428)	F	432	R	FD, B_Ia, B_Ib, B_II
141 (141)	F	142	T	FD	473 (430)	E	434	V	B_II
143 (143)	L	144	T	B_Ia	496 (453)	G	458	Q	FD, B_II
146 (146)	A	147	P	B_Ia	497 (454)	E	459	G	B_II
161 (161)	S	162	K	B_Ib	498 (455)	D	460	A	B_II
162 (162)	Q	163	D	FD	508 (465)	N	470	K	B_II
170 (170)	I	174	I	B_Ib	510 (467)	R	472	N	B_Ia, B_Ib, B_II
189 (189)	S	193	L	B_Ia	512 (469)	Q	474	H	B_Ib, B_II
192 (192)	G	196	K	B_Ia	517 (474)	T	479	V	FD
203 (203)	D	207	L	B_II	518 (475)	E	480	E	FD, B_Ia
204 (204)	S	209	P	B_Ib	526 (483)	S	488	S	B_Ia
218 (218)	S	223	D	B_Ib	585 (542)	Y	542	S	B_Ib, B_II
222 (222)	L	227	E	B_II	586 (543)	H	543	T	FD, B_Ia, B_Ib
224 (224)	D	229	P	B_Ia, B_Ib	588 (545)	T	545	E	B_Ia, B_Ib
248 (248)	E	253	A	B_II	593 (550)	K	547	S	B_Ib
266 (266)	G	271	K	B_Ia, B_Ib, B_II	595 (552)	L	549	D	B_Ia, B_Ib, B_II
274 (274)	M	279	Q	FD	614 (571)	D	569	S	B_Ib
322 (279)	D	284	V	B_Ib	618 (575)	H	573	S	FD, B_Ib
332 (289)	A	294	D	B_Ia, B_Ib	623 (580)	S	578	R	FD, B_Ib
337 (294)	S	299	L	B_II	626 (583)	D	581	R	B_Ia
343 (300)	M	305	Q	PS	639 (596)	Q	594	T	FD
351 (308)	R	312	Y	B_II, PS	640 (597)	S	595	I	B_Ib
356 (313)	R	317	L	B_II	641 (598)	E	596	S	FD, B_Ib
357 (314)	L	318	P	B_Ia, B_Ib, PS	657 (614)	H	610	A	B_Ia, B_II, PS
381 (338)	G	342	E	B_II	703 (660)	Y	656	S	FD, B_Ia, B_Ib
383 (340)	H	344	I	FD, B_Ia, B_Ib	717 (674)	E	670	W	B_Ib

FunDi aims to detect sites under functional divergence [58], independent of whether they belong to Type I or Type II of functional divergence. A stringent 95% threshold of posterior probability was used. FunDi requires a MSA without gaps (insertion or deletion). As some of the sequences contained deletions or had ambiguous residues (annotated with multiple ‘X’), 30 different MSAs were generated by removing one sequence at a time, and the analyses were performed on all these alignments. This ensured a greater coverage than focusing on the whole alignment.

BADASP and FunDi were applied to all these alignments and every detected site was retained.

### 3.4. Identification of codons under positive selection

A change in amino acid can promote a functional change that can be ultimately adaptive. This new adaptive change will then be retained by positive Darwinian selection. The detection of such positive selection at the residue level in protein can be inferred by the estimation of the number of non-synonymous (dN) substitutions, which change the coded amino acid, and the number synonymous (dS) substitutions, which do not change the coded amino acid. A dN/dS ratio can be computed to estimate the selective pressure acting on that gene, and a ratio > 1 is an indicator of positive selection, while a ratio < 1 is an indicator of negative (purifying) selection. A dN/dS ratio close to 1 indicates that the gene is evolving neutrally. A statistical branch-site model that tends to identify positive selection that happened on a subset of sites (codons) in a specific lineage (branch) is implemented in the CodeML/PAML package [60,61]. Positive selection on a specific branch is then identified by a likelihood-ratio test (LRT) based on a null-model that does not allow positive selection (only neutral and negative selection) versus a model that allows positive selection (and neutral and negative selection). When the LRT is significant, after correction for false-discovery rate, codons that contribute to this positive selection can be identified by a Bayes empirical Bayes (BEB) test. Sites can be classified under relax and strict thresholds of BEB score > 0.95 and BEB score > 0.99, respectively.

The codons under positive selection between GAS6 and PROS1 were retrieved from the Selectome database of precomputed tests of positive selection [62,63], which uses the branch-site model from CodeML/PAML. In Selectome, we focused solely on the branch named ‘Euteleostomi’ (which corresponds roughly to the basis of vertebrates, as sharks and sea lampreys are not present in Selectome), which separated the paralogous genes PROS1 and GAS6.

### 3.5. Stability effect

The contribution of residues to the SHBG-domain stability was computed by FoldX [64], using the function ‘build model’. We choose the crystallized SHBG domain of GAS6 (PDB ID: 2C5D) because it is the main domain of the protein and the one participating in interaction. Each amino acid has been mutated to itself to estimate its contribution to the energy of the wild-type (Δ*G*_wt_, in kcal mol^−1^). Second, each amino acid is mutated to all the other 19 amino acids, to calculate the energy of the mutant (Δ*G*_mut_, in kcal mol^−1^). Therefore, the difference between Δ*G*_wt_ and Δ*G*_mut_ was calculated to give the value for ΔΔ*G* (=Δ*G*_mut_ − Δ*G*_wt_), the stability effect of replacement of one amino acid for another. The final result was a substitution matrix for all the amino acid positions in the GAS6 protein structure.

### 3.6. Electrostatic surface analysis

The human structures of GAS6 and PROS1 were modelled by homology using Modeller [65]. The template used was the SHBG-domain of GAS6 (PDB ID: 2C5D). We decided to model the GAS6 over its crystallized structure, in order to facilitate the direct comparison with the model of PROS1. The electrostatic surfaces were computed using APBS (Adaptive Poisson–Boltzmann Solver), a suite for performing Poisson–Boltzmann electrostatic calculations on biomolecules [66] and visualized in PyMOL [67].

### 3.7. Prediction of protein–protein interactions

GAS6 residues (PDB ID: 2C5D) involved in protein–protein interactions were predicted with the Optimal Docking Area (ODA) program from the ICM Pro package (Molsoft) [68].

## 4. Results and discussion

### 4.1. Data collection

Representative sequences were collected using BlastP searches of SHBG, PROS1 and GAS6 amino acid sequences against the UniProtKB/Swiss-Prot database (www.uniprot.org). These sequences were aligned with MAFFT [52]. Within each PROS1, GAS6 or SHBG clade, the sequences share between 100 and 50% pairwise identical residues, respectively. However, only approximately 40% identical residues were found between the two clades of, respectively, GAS6 and PROS1 sequences, and sequences of the SHBG clade share only between 22 and 28% of residues with sequences of the GAS6 and PROS1 clades, respectively.

### 4.2. Phylogenetic analysis

Phylogenetic inference using NJ, ML and Bayesian analysis resulted in three almost identical and very robust trees (with high confidence score per node). The phylogenetic tree clearly showed three separate clades representing the SHBG, the PROS1 and the GAS6 clusters (figure 1). Although unrooted, it is clear that the SHBG clade may serve as an outgroup to the other two clades present in the tree. The tree topology within the PROS1 and GAS6 clusters was identical. The evolution of the three genes can be explained by two rounds of whole-genome duplications (proposed by Ohno [69] and reviewed in [47] and [70]), where the first event of duplication of an ancestral gene led to the formation of the *SHBG* gene and the ancestor of *PROS1*/*GAS6* genes, while a second and later duplication event resulted in the formation of separate *PROS1* and *GAS6* genes. The similarity of the branching order within the latter two clades represents the events of speciation that took place during vertebrates' evolution. To obtain further information about the time scale at which the various events took place, a relaxed clock model was applied using the MrBayes program. First, using the estimated dates for the divergence of various taxa available in the TimeTree database (figure 1; electronic supplementary material, table S1) [56], the median clock rate was estimated to be 0.00136 amino acid substitutions per site per million years (figure 1; electronic supplementary material, table S1). Using this rate, the evolutionary times of the two duplication events were calculated to be 697 million years ago (Ma) for SHBG/PROS + GAS6 and 649 Ma for PROS/GAS6 (figure 1; electronic supplementary material, table S1). These values are in line with the evolution of vertebrates, as the split between vertebrates and the urochordate *Ciona* is estimated around 700–800 Ma, according to TimeTree.

### 4.3. Structural impact of the mutations

The replacement of one amino acid by another may have an effect on protein structure, depending on the position and physico-chemical properties of the substitution. Some changes can be very drastic; for example, the replacement of a hydrophobic residue such as alanine with a charged residue such as glutamic acid within the core of a protein structure is likely to have major consequences for the local and overall packing of amino acid residues. In the case of a functional divergence between proteins, it has been hypothesized that amino acid replacements tend to be more divergent (stabilizing or destabilizing) compared with amino acids evolving under a neutral process, where the function is preserved [71]. This has previously been observed in a dataset of 22 different enzymes [72], as well as in ribulose-1,5-bisphosphate carboxylase/oxygenase (RubisCO) [73] and in cetacean myoglobins [74].

**Figure 2.**
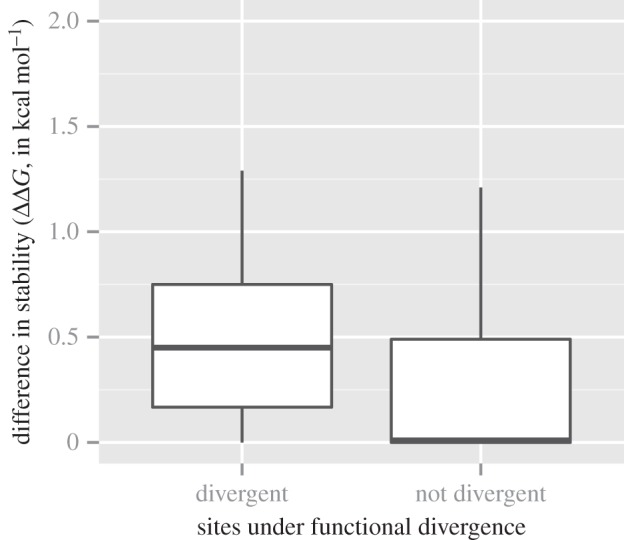
Comparison of the stability effect between sites under functional divergence and other sites. The *x*-axis represents the categories of sites detected by FunDi, BADASP or Selectome. The *y*-axis represents the absolute median difference in stability effect (ΔΔ*G*, expressed in kcal mol^−1^) between the group of amino acids in PROS1 versus the group of amino acids in GAS6. These values were estimated with FoldX based on the structure of GAS6 (PDB ID: 2C5D) [22]. Values above 0.5 kcal mol^−1^ are slightly destabilizing, above 1 kcal mol^−1^ are destabilizing and above 2 kcal mol^−1^ are strongly destabilizing.

To estimate the effect of an amino acid replacement, we first calculated the effect on protein stability (ΔΔ*G*, in kcal mol^−1^) for all residue replacements in the GAS6 structure (PDB ID: 2C5D [22]). Each residue was mutated *in silico* to all 19 other amino acids and the ΔΔ*G* was recorded. We calculated the composition of amino acids in each column of the MSA, and using this we defined the contribution of each amino acid for each sequence to the protein structure stability. Using the matrix thus generated, we estimated the median ΔΔ*G* (in kcal mol^−1^) for each column of GAS6 and for each column of PROS1. Then, we computed the difference in stability (in absolute value) between GAS6 and PROS1. The mean and median of all the 391 differences were 0.68 and 0.15 kcal mol^−1^, respectively. We then separated these positions between sites under functional divergence and sites not under functional divergence (figure 2). The mean of non-divergent sites was 0.56 kcal mol^−1^, while the mean of divergent sites was 1.45 kcal mol^−1^. Similarly, the median of non-divergent sites was 0.08 kcal mol^−1^, while the median of divergent sites was 0.60 kcal mol^−1^. The difference between sites under functional divergence and sites not under functional divergence was significant (Wilcoxon signed-rank test, *p*-value = 1.932 × 10^−8^). This would support the above-mentioned hypothesis that sites under functional divergence have a greater effect on the protein structure than sites not detected to be under functional divergence.

### 4.4. Three-dimensional visualization

#### 4.4.1. General

**Figure 3.**
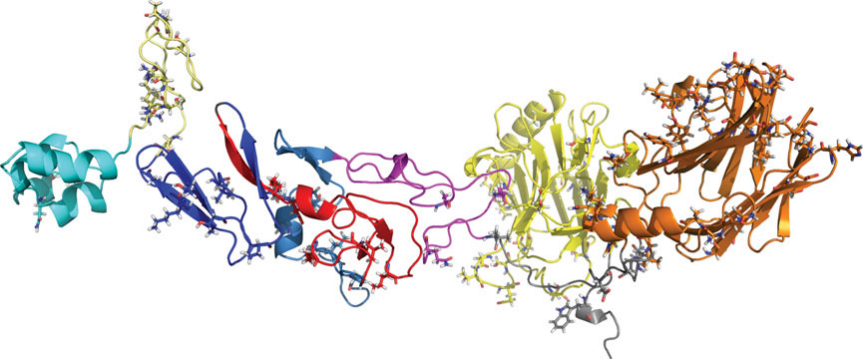
Global view of all sites on PROS1. This is a composite model of the whole PROS1 using different templates. The modelling has been done with Yasara What If. Colouring is domain specific: GLA (cyan), TSR (light yellow), EGF1 (dark blue), EGF2 (red), EGF3 (slate), EGF4 (mangenta), LG1 (yellow) and LG2 (orange).

**Figure 4.**
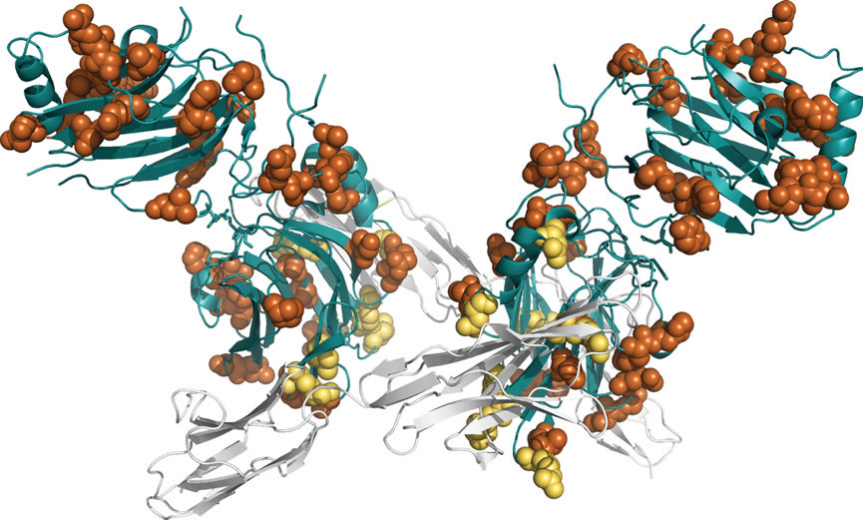
Three-dimensional visualization of GAS6 in complex with Axl (PDB ID: 2C5D). Sites under functional divergence are shown as spheres and coloured in orange. Sites under functional divergence and in contact with Axl (in cartoon and in white) are in yellow. *α*-helices and *β*-sheets of GAS6 domain are in blue.

**Figure 5.**
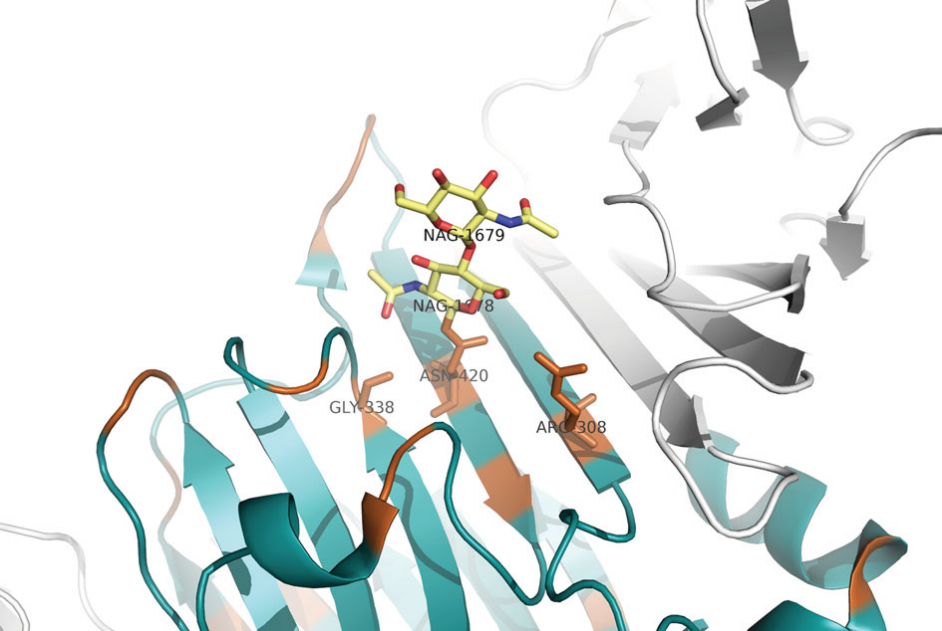
Visualization of *N*-acetylglucosamine (NAG) binding site. The asparagine at position Asn463(420) in GAS6 is mutated to a lysine in PROS1. NAG ligand is in stick and coloured in yellow. Sites under functional divergence are coloured in orange. *α*-helices and *β*-sheets of GAS6 domain are in blue. Axl domains are in grey.

We used the crystallized structural complex of Gas6–Axl (PDB ID: 2C5D [22]) and a homology-based structure for PROS1 to visualize the location of sites under functional divergence (figures 3–5). Residual numbering for GAS6 is based on isoform 1 or 2 (between brackets). We followed the Human Gene Variation Society (HGVS) numbering where the methionine encoded by the translation initiation site is numbered as residue 1 [75].

### 4.5. Residues close to Axl

Nine sites under functional divergence were present at the interface with Axl: Met343(300), Arg351(308), Arg356(313), Leu357(314), Val435(392), Arg445(402), Arg457(414), Asp498(455) and Asn508(465) (figure 4). Only residue Met343 was detected by Selectome as being under positive selection. The methionine in GAS6 was replaced by a glutamine in PROS1. Val435 and Arg457 are very closely located in the three-dimensional structure, and Arg457 directly in contact with Axl. The hydrophobic Val435 is mutated into a polar Ser396 in PROS1, and a polar positively charged Arg457 is mutated into polar uncharged Asn418, except in rodents, where there is an aspartate (another negatively charged amino acid). Asp498 and Asn508 are on both sides of the helix.

### 4.6. Residues that have been reported to be involved in binding of PROS1 to C4BPβ

Four sites under functional divergence are present at the interface (less than 6 Å) with C4BPβ: Lys470, Asn472, His474 and Ser488. In the three-dimensional structure of PROS1 Lys470, Asn472 and His474 are sequentially clustered on LG1, whereas Ser488 is situated on LG2 mirrored to Asn472 at an estimated distance of 9.2 Å. The basic residues Lys470 and His474 are mutated to the polar residues Asn508(465) and Glu512(469) in GAS6, respectively. The polar Asn472 is mutated to the basic Arg510(467).

### 4.7. Residues that have been reported to be involved in binding of PROS1 to FVa

One site (residue 670) under functional divergence is present at the binding site for FVa [35]. The hydrophobic residue Trp670 in PROS1 is mutated to the charged acidic residue Glu717(673) in GAS6.

### 4.8. Residues close (less than 6 Å) to *N*-acetyl-d-glucosamine

GAS6 and PROS1 are both post-translationally modified through *N*-linked glycosylation, but at different amino acid positions. *N*-linked glycosylation occurs at the attachment site (or sequon), whose consensus sequence is Asn-X-Ser/Thr (N-X-S/T), where the *N*-glycans are covalently attached to the protein at an asparagine (Asn) residue. *N*-glycans typically contain three mannose residues and two *N*-acetylglucosamine (NAG) residues, where NAG is directly linked to the asparagine side chain.

In GAS6, *N*-glycosylation occurs at one residue in the first Laminin G-like domain (LG1), at asparagine Asn463(420), which is on a β-strand in the core of the structure (figure 5). This asparagine is detected to be under Type II of functional divergence (replaced by a lysine in PROS1). The residue at position *n* + 2 is a threonine, which is also under Type II of functional divergence (replaced by a tyrosine in PROS1). These two changes break the N-X-S/T motif and prevent *N*-glycosylation at this sequon. Two other sites in contact (less than 6 Å) with NAG were also detected to be under functional divergence: Arg351(308) and Gly381(338). They are on different β-strands and replaced by a tyrosine and a glutamic acid in PROS1, respectively. Similarly, some residues, which are part of the pocket where NAG is located, are under functional divergence. For example, Thr465(422) is in the same β-strand as Asn463(420). Gln384(341) is in close contact with Phe471(428), which precedes His429. Gln384(341) is replaced by an aspartate in PROS1 and Phe471(428) by a lysine.

In PROS1, *N*-glycosylations occur at three asparagine residues (Asn499, Asn509 and Asn530) in the second Laminin G-like domain (LG2). These three residues were not detected to be under functional divergence, but they were also different in GAS6, where they were replaced by Arg537 and Glu551. Asn530 is an insertion in PROS1 primates. The region around Asn530 is very divergent and none of the residues around Asn530 in PROS1 were found to be functionally divergent. However, residues Arg578, Arg581 and Ala610, which are in close three-dimensional proximity (8 Å) to the NAG binding sites Asn499, Asn509 and Asn530, respectively, were found to be functionally divergent. In GAS6, these residues were replaced by Ser623(580), Asp626(583) and His657(614), respectively (electronic supplementary material, figure S1).

### 4.9. Comparison of electrostatic surfaces between PROS1 and GAS6

**Figure 6.**
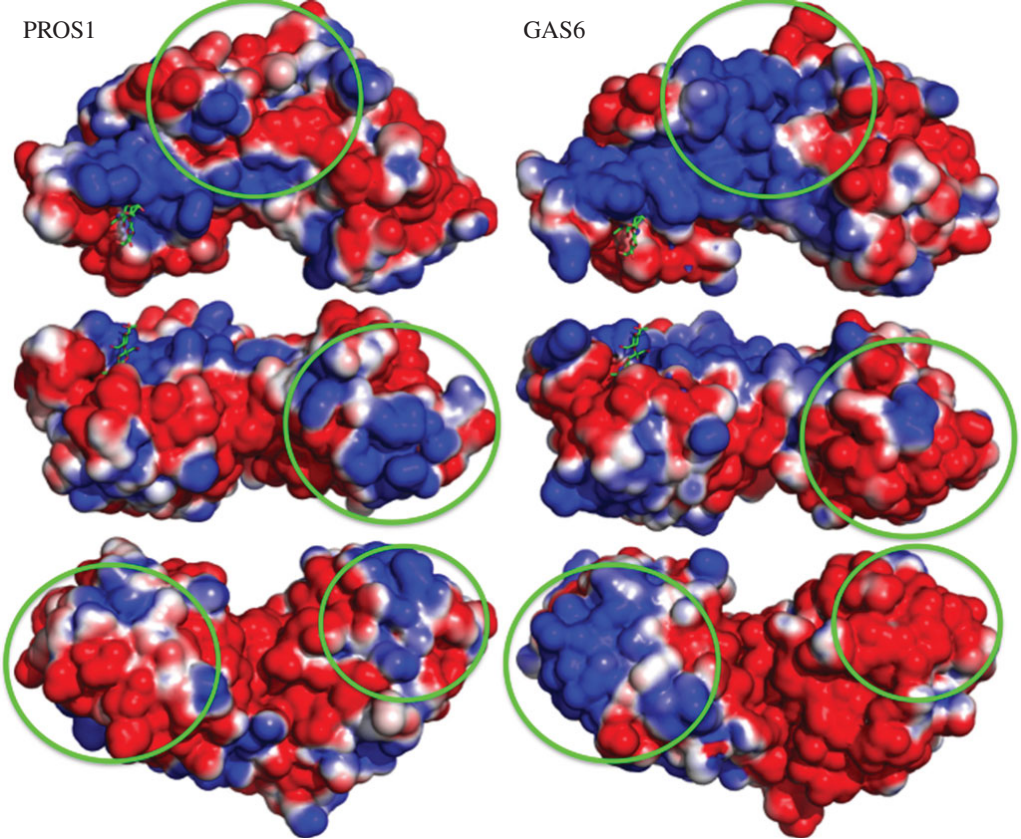
Visualization of electrostatic surfaces on the SHBG-domain of PROS1 and GAS6. To make the direct comparison between GAS6 and PROS1 easier, we have modelled their SHBG domains using the GAS6 PDB structure (PDB ID: 2C5D). NAG ligand has been added to identify its putative binding pocket. While it is crystallized in GAS6, there is no evidence to indicate whether it can be present in PROS1. Basic surfaces are in blue while acidic surfaces are in red. The NAG ligand is in green. The green circles indicate the observed differences in electrostatic surface potential between GAS6 and PROS1.

Using APBS [66] to compute the electrostatic properties of the surfaces of the SHBG domain, we observed three patches that are different between PROS1 and GAS6 (figure 6).

The first patch showed a strong basic patch in GAS6 (in blue), formed by residues Ala332(289), Lys333(290), Lys336(293), Lys506(463), Arg510(467) and Arg684(641), while in PROS1, the corresponding residues were either polar or acidic (Asp294, Thr295, Glu298, Gln468, Asn472 and Asn637). Residues Ala332(289) and Arg510(467) in GAS6 and residues Asp294 and Asn472 in PROS1 have been detected to be under functional divergence. On this patch residues Gln468 and Asn472 were located in the binding site of C4BPβ.

The second patch is more acidic in GAS6, formed by Arg537(494), Ser623(580), Asp626(583), Glu628(585), Gln648(605), Glu649(606), Arg656(613) and Arg659(616), while more basic in PROS1 (Asn499, Arg578, Arg581, Asn583, Gln601, Arg602, Lys609 and Lys612). Residues Ser623(580) and Asp626(583) in GAS6 and residues Arg578 and Arg581 in PROS1 on this patch were detected to be under functional divergence. Asp499 in PROS1, attached to NAG, is located at the binding site of C4BPβ.

The third patch is basic in GAS6 formed by Arg403(380), Asn405(362), Ala431(388), Arg432(389), Lys445(402) and Ala447(404), while it is an acidic patch in PROS1 formed by Lys364, Glu366, Glu392, His393, Asp406 and Asn408. Residues Asn405(362), Arg432(389) and Lys445(402) in GAS6 and residues Glu366 and Asp406 in PROS1 were detected to be under functional divergence. This patch is at the contact interface between GAS6 and Axl.

### 4.10. Protein–protein interaction prediction

**Figure 7.**
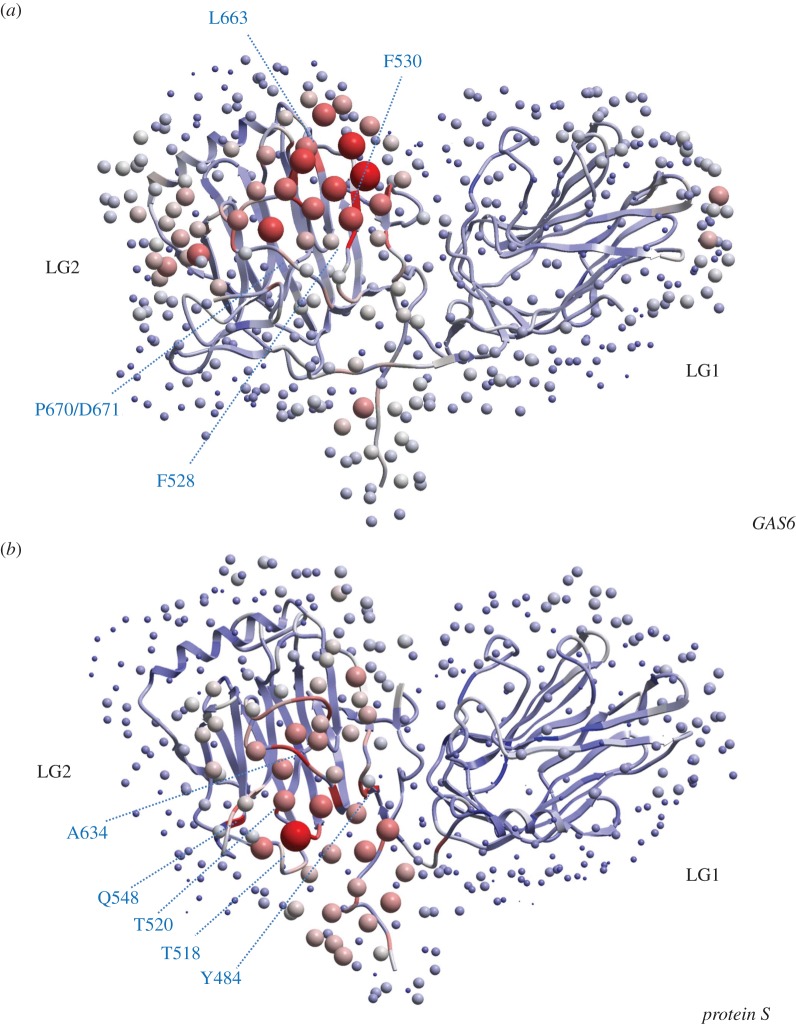
Optimal docking area analysis for LG1 and LG2-domains of GAS6 and PROS1. Red dots indicate likely interaction areas, blue indicates protein–protein interactions to be unlikely. (*a*) For GAS6, the most likely interactions are with F528, F530, L663, P670 and D671. (*b*) For PROS1, the most likely interactions are with Y484, T518, T520, Q548 and A634.

We used the ODA analysis [68] to identify residues that may be responsible for protein–protein interactions. ODA works essentially for protein–protein interaction predictions that involve large hydrophobic patches. ODA identifies optimal surface patches with the lowest docking desolvation energy values as calculated by atomic solvation parameters (ASP) derived from octanol/water transfer experiments and adjusted for protein–protein docking. Using the ODA analysis, we identified residues that may be responsible for protein–protein interactions (figure 7).

We observed a striking difference between the two proteins and a clear shift in the predicted protein–protein interaction areas between the two proteins. Note that these residues are present in the LG2 domain of both proteins. Three distinct regions within the LG2 domain of PROS1 have been identified to interact with C4BP (488–501 and 646–655) [25–27,31,32] and FVa (662–676) [35]. No residues were located in one of these three regions. For GAS6, the interactions with Axl in the 2C5D structure appear exclusively mediated through LG1 [22]. Potentially, the predicted residues of GAS6 mediate contact to other receptors such as Mer or Tyro3, all of which have been shown to interact with GAS6. However, among the identified residues within the LG2 domain of GAS6, Phe528 is suggested to have a controlling function in GAS6–Axl interactions [22]. None of the predicted residues was predicted to be under functional divergence.

## 5. Conclusion

GAS6 and PROS1 have been widely studied for their biological functions. Despite their homology and structural resemblances, both proteins exhibit distinct functions. In this study, we investigated the evolutionary trajectory of the paralogous genes *GAS6* and *PROS1* to better understand how these two genes became functionally different. Our results indicate that these genes emerged at the beginning of vertebrate evolution, which is estimated at 550–700 Ma, since the last split with urochordates (like the tunicate *Ciona intestinalis*). This also corresponds to the time when the two rounds of whole-genome duplication occurred in vertebrates. Additionally, we identified residues under functional divergence in the two proteins encoded by *GAS6* and *PROS1*. These residues were scattered throughout the two genes. However, approximately 60% of all residues under functional divergence were located in the SHBG domain (LG1/LG2) in both the proteins. GAS6 and PROS1 require this domain for their distinct functions. Only a small fraction of functionally divergent residues were located in the binding site. We also determined the implications of the sites under functional divergence on the structures of GAS6 and PROS1. From these data, we conclude that the sites under functional divergence are predominantly required for the overall structure and function of both proteins. We identified functionally important sites, which will help in understanding the molecular basis of the functional divergence between both these genes as well as providing significant information about species-specific adaptation. Finally, these results might help researchers to analyse disease-causing mutations in the light of evolution and structural constraints.
